# Transparent Organic Photodetector using a Near-Infrared Absorbing Cyanine Dye

**DOI:** 10.1038/srep09439

**Published:** 2015-03-24

**Authors:** Hui Zhang, Sandra Jenatsch, Jelissa De Jonghe, Frank Nüesch, Roland Steim, Anna C. Véron, Roland Hany

**Affiliations:** 1 Empa, Swiss Federal Institute for Materials Science and Technology, Laboratory for Functional Polymers, CH-8600 Dübendorf, Switzerland; 2 Photochemical Dynamics Group, Institute of Chemical Sciences and Engineering, Ecole Polytechnique Fédérale de Lausanne, EPFL, Station 6, CH-1015 Lausanne, Switzerland; 3 Institut des Matériaux, Ecole Polytechnique Fédérale de Lausanne, EPFL, Station 12, CH-1015 Lausanne, Switzerland

## Abstract

Organic photodetectors are interesting for low cost, large area optical sensing applications. Combining organic semiconductors with discrete absorption bands outside the visible wavelength range with transparent and conductive electrodes allows for the fabrication of visibly transparent photodetectors. Visibly transparent photodetectors can have far reaching impact in a number of areas including smart displays, window-integrated electronic circuits and sensors. Here, we demonstrate a near-infrared sensitive, visibly transparent organic photodetector with a very high average visible transmittance of 68.9%. The transmitted light of the photodetector under solar irradiation exhibits excellent transparency colour perception and rendering capabilities. At a wavelength of 850 nm and at −1 V bias, the photoconversion efficiency is 17% and the specific detectivity is 10^12^ Jones. Large area photodetectors with an area of 1.6 cm^2^ are demonstrated.

Photodetectors are present in a vast variety of devices used in private households, industry and research. Organic photodetectors may be well-suited for applications that require large active area, mechanical flexibility, low-cost processing, or wavelength specificity^1^. Applications where organic photodetectors with a narrow spectral response in the near-infrared (NIR) wavelength range would be beneficial are remote control, reflective sensors such as optical communication^1^. Another example can be found in the field of biochemical sensing. A fluorescent molecule can serve as a reporting group when tagged to a biomolecule. When using NIR fluorescent dyes, the background noise caused by the auto-fluorescence of the biosubstrate can be greatly reduced. In addition, the high tissue penetration of NIR light enables *in vivo* imaging applications^2^.

Multicolour organic photodetectors with sensitivity extending to the NIR wavelength range have been presented using low band gap polymers^3^,^4^ or small molecules^5^,^6^,^7^. When exclusive NIR light sensing is required, these photodetectors must be operated using a visible light filter. Recently, organic photodetectors with selective NIR sensitivity have been reported^8^,^9^,^10^. These detectors used squaraines and J-aggregated cyanine small molecules with narrow and intense absorption features in the wavelength range from ~650–850 nm.

Combining organic NIR dyes with transparent, non-reflective electrodes allows for the fabrication of visibly transparent devices, thereby adding new functionality to organic photodetectors, such as integration in displays or invisible electronic circuits^11^. (Semi-)transparent organic solar cells are intensively being investigated for applications such as building-integrated photovoltaics or chargers of portable electronics^12^,^13^, but only few attempts were made to demonstrate transparent organic photodetectors. A visibly transparent photoconductor was reported using a naphthalocyanine molecule with an absorption maximum at ~1000 nm^14^. Semitransparent photodetectors with an average visible transmittance (AVT) of 45% were fabricated with molecules that absorb strongly in the ultraviolet and NIR region but relatively weakly in the visible^6^. A stacked device was reported that consisted of an organic light-emitting diode (OLED) and a semitransparent (AVT ~ 45%) photodiode^15^. The device acts as an image sensor by emitting light from the OLED through the photodiode onto a surface and detecting reflected light by the photodiode.

## Results and Discussion

**Figure 1:**
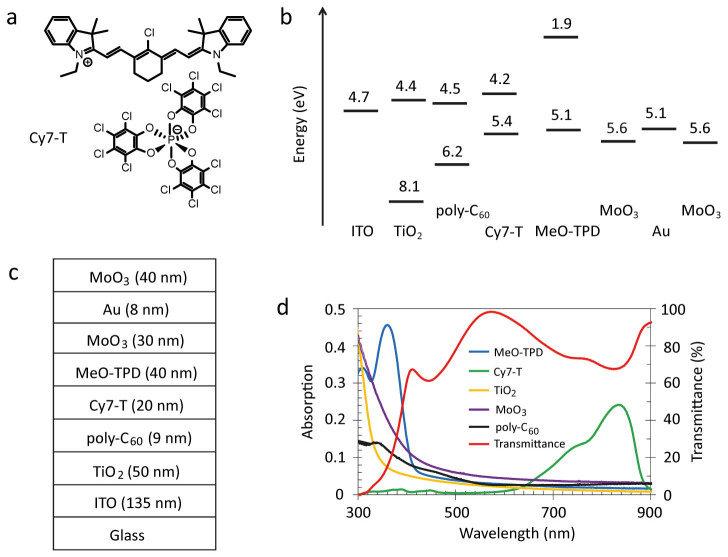
Molecular structure of the cyanine dye, schematic of the device architecture and absorption spectra. (a) Chemical structure of the cyanine dye Cy7-T with the anion Δ-TRISPHAT. (b) Energy level diagram of the photodiode. (c) Schematic representation of the photodiode with an average visible transmittance (AVT_450–670 nm_) of 68.9%. (d) Absorption spectra of individual materials with thicknesses indicated in (c), and the transmittance spectrum of the layer stack ITO/TiO_2_/poly-C_60_/Cy7-T/MeO-TPD/MoO_3_.

Here, we demonstrate a sensitive, fast responding and transparent organic photodetector with a high average visible transmittance of over 65%. The transmitted light of the photodetector under solar irradiation exhibits excellent transparency colour perception and rendering capabilities. The photodetector consists of a TiO_2_ electron transport layer^16^ on ITO that is sensitized by a near-infrared absorbing heptamethine cyanine dye layer, Cy7-T (Figure 1a). Recently, we fabricated transparent organic photovoltaic cells using Cy7-T with an average visible transmittance of 66% and a power conversion efficiency of 2.2%^17^. The device further contains poly-C_60_ (photo-polymerized C_60_) and MeO-TPD (N,N,N′,N′-Tetrakis-(4-methoxyphenyl) benzidine) interfacial layers that are inserted to reduce the dark current (Figure 1b and 1c).

**Figure 2:**
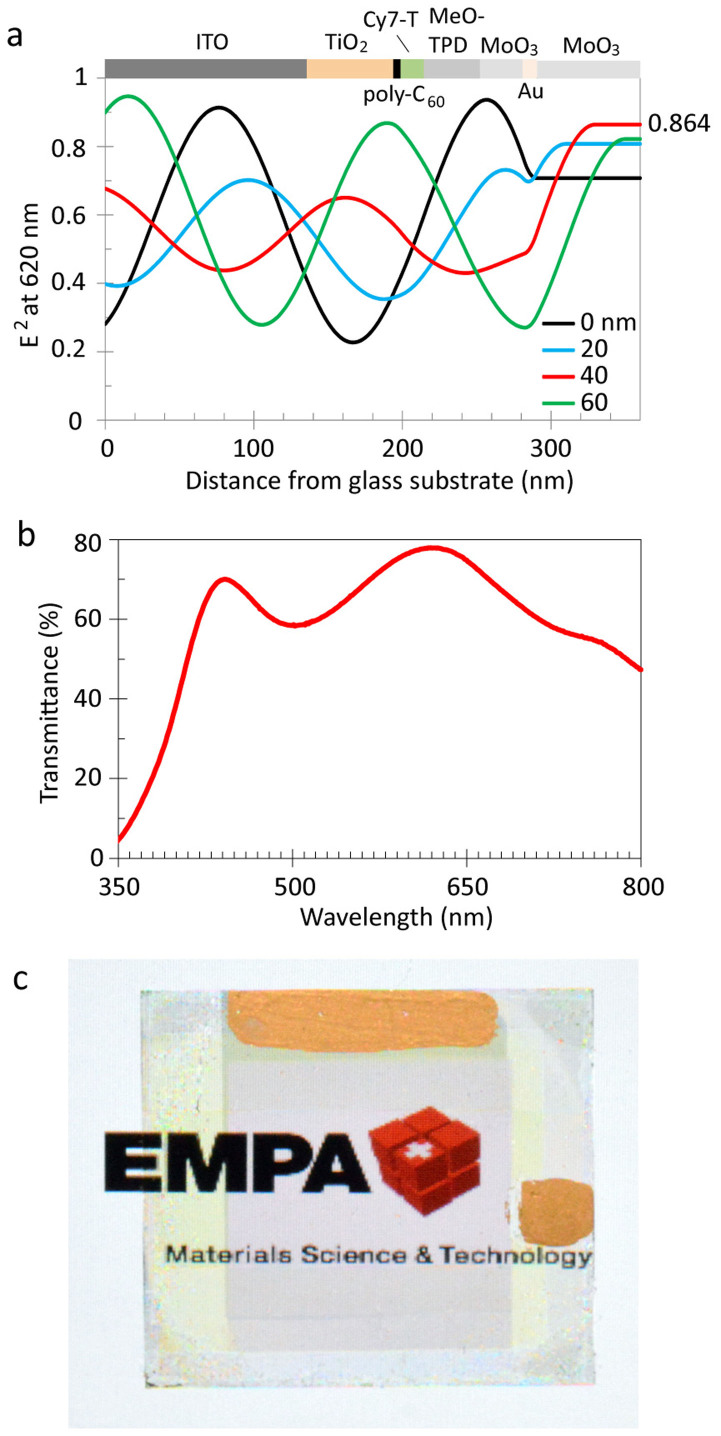
Optical device properties. (a) Calculated spatial distribution of the normalized squared optical electric field strengths for λ = 620 nm inside transparent photodetectors. (b) Transmittance spectrum and (c) image of the transparent photodetector with an active area of 1.6 cm^2^. The EMPA logo is reproduced with permission from the Swiss Federal Institute for Materials Science and Technology.

Light absorption in the visible is small for all materials and the transmittance of the multilayer stack, excluding the top electrode, is above 90% between 530 and 640 nm (Figure 1d). Photodetectors were fabricated with either non-transparent 80 nm thick Ag anodes or a semitransparent Au/MoO_3_ top contact (Figure 1c). Gold was the hole-collecting electrode and MoO_3_ an additional external dielectric coating to increase the device transmittance. Wavelength-dependent optical modelling was carried out for different Au and MoO_3_ film thickness combinations to simulate full transmittance spectra from which calculated average visible transmittance values were extracted. For pure Au layers, AVT values increased monotonically with decreasing thickness. However, a balance must be achieved between optical transparency and electrical conductivity (Supplementary Information S1). A low electrode sheet resistance is important for photodetector scale-up, in order to reduce losses in transporting charge to external circuitry. To ensure that Au is forming a continuous film in a reproducible way the gold thickness was fixed to 8 nm (AVT_390–720 nm, calc._ = 64%)^18^. AVT values increased to a maximum of AVT_390–720 nm, calc._ = 71.5% when adding 40 nm MoO_3_ on top. The optical electric field distribution inside the device varies considerably by changing the MoO_3_ thickness. As an example, Figure 2a shows simulated normalized spatial distributions of the squared electric field strengths at λ = 620 nm for MoO_3_ layer thicknesses ranging from 0 to 60 nm. The strength of E^2^ leaving the device corresponds directly to the transmittance. For a 40 nm thick MoO_3_ layer, the calculated transmittance was 86.4% at λ = 620 nm, in fair agreement with the experimental value of 77.8% (Figure 2b).

The value of the AVT of the photodetector depends on the definition of the visible wavelength range. Common assessments for the visible range are defined for photopic responses >0.1% or >5% peak sensitivity, resulting in visible spectral ranges of ~390–720 nm and ~450–670 nm, respectively^19^. For these wavelength ranges, experimental average visible transmittance values are AVT_390–720 nm, exp._ = 66.4% and AVT_450–670 nm, exp._ = 68.9%.

The photodetector exhibits a very high and uniform measured transmittance over a large range of the visible spectrum, resulting in a greyish and colour neutral appearance (Figure 2c). There are several ways how the measured transmitted light can be related to the human perception of transparency and colour. Since the sensitivity of the human eye is different for every visible wavelength, the human perception of transparency (HPT) can differ from the radiometric AVT value. To calculate HPT, the measured transmittance is folded with the eye sensitivity^20^. A HPT_390–720 nm_ value of 68.4% was obtained, in good agreement with the AVT value. For analyzing the transparency colour perception, colour coordinates were calculated in the CIE 1931 colour space. As light source, we used the AM1.5 solar spectrum folded with the transmittance spectrum of the photodetector^21^. Colour coordinates were (0.350, 0.342), very close to the so called “white point” (1/3, 1/3). This implies that the transparency colour perception is similar to the colour perception of the original light source and the device is acting nearly as a neutral density filter.

The colour rendering index (CRI) is a quantitative measure for how well a light source, in our case the transmitted light from the photodetector, renders the colours of objects in comparison to a reference light source. CRI was evaluated following published procedures^21^,^22^. Therefore, the special colour rendering index (sCRI) was calculated for eight standard test color samples. The correlated colour temperature of the photodetector was T = 4760 K and the blackbody irradiator was used as the reference light. A CRI of 100 means that the eight specified colours have the same appearance when illuminated by the transmitted light from the photodetector or by the reference light source, by definition. We obtained eight sCRI values (96, 96, 98, 98, 96, 95, 98, 98) and from these, a general (i.e. the mean) CRI of 96.9. This high general CRI implies an excellent colour rendering capacity that is comparable with the highest reported CRI values for semitransparent organic solar cells^22^.

**Figure 3:**
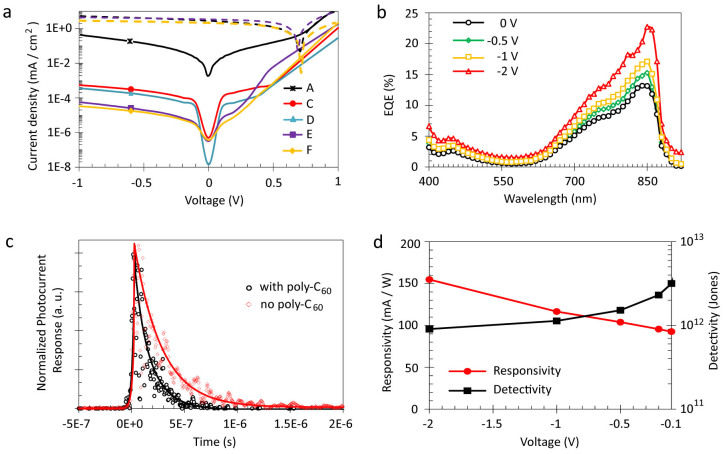
Electrical device properties. (a) Current-voltage (J–V) characteristics of photodetectors measured in the dark (solid lines) and under 1 sun illumination (dashed lines). Labels designate devices shown in Table 1. (b) External quantum efficiency versus wavelength for a transparent photodetector (device F) at various biases. (c) Photocurrent response of photodetectors (device F) to a 5 ns long light pulse at 850 nm. (d) Responsivity and specific detectivity at 850 nm of photodetector F for different applied biases.

To tune the electrical properties of the photodetector, we omitted in a first step the interfacial layers and fabricated TiO_2_/Cy7-T heterojunction devices (Table 1, device A). Operated in the photovoltaic mode under 1 sun illumination, the short-circuit current was J_sc_ = 2.7 mA cm^−2^, the open-circuit voltage V_oc_ = 0.74 V, and the fill factor FF = 37%, resulting in a power conversion efficiency of η = 0.7% (Figure 3a). This confirms the electron transfer process from the photoexcited dye into the TiO_2_ conduction band, and the regeneration of the Cy7-T ground state by hole transfer across MoO_3_.

However, the dark current (J_d_ = 4.3 × 10^−1^ mA cm^−2^ at −1 V) is too high for using device A as a photodetector. The dark current is an inherent source of detector noise, and the entity of noise determines the lower limit of light detection. In general, applying a reverse bias across a photodetector increases its speed of response. However, the dark current tends to increase as well with applied reverse bias resulting in an increase of the shot noise. Therefore, the choice of operation mode is a trade-off between the required speed of response and the maximum noise that can be accepted in the actual application.

A major contribution to the dark current in organic photodetectors under reverse bias is the injection of charge carriers through the electrode contacts into the semiconductor materials^1^,^9^,^23^. A proven strategy to suppress these injection processes is the use of interfacial blocking layers^1^,^4^,^9^. We selected MeO-TPD as suitable electron blocking layer (Supplementary Information S2). MeO-TPD does not absorb light in the visible and has a high LUMO energy to block electron injection. In addition, the HOMO energies of MeO-TPD and Cy7-T closely match, which is essential for efficient photogenerated carrier collection. TiO_2_/Cy7-T/MeO-TPD devices with increasing thickness of MeO-TPD were fabricated (Table 1, B–D). From spectral response measurements, we observed no photocurrent generation in the wavelength range below 400 nm where MeO-TPD absorbs light. This means that MeO-TPD acts solely as an electron blocking and hole transporting layer. The optimum layer thickness of 40 nm MeO-TPD resulted in a dark current reduction by three orders of magnitude (J_d_ = 5.5 × 10^−4^ mA cm^−2^ at −1 V; Table 1, C) while reducing J_sc_ from 2.73 mA cm^−2^ for device A to 1.70 mA cm^−2^ only slighty. The dark current decreased further by increasing the MeO-TPD thickness to 100 nm, but J_sc_ collapsed due to the strong increase in the series resistance (Table 1, D).

A C_60_ film was evaporated onto TiO_2_ and photo-polymerized by illumination (Supplementary Information S3)^24^. Poly-C_60_ is partially insoluble in the solvent chlorobenzene that was used to spin coat the Cy7-T film on top. In agreement with results from related Cy7/C_60_ photovoltaic cells, we observed a strong increase of the forward injection current under illumination compared to the dark current (Figure 3a)^17^,^25^. This photoconductivity effect^26^ probably originates from light absorption in the C_60_ layer^27^,^28^. Insertion of the C_60_ interfacial layer between TiO_2_ and Cy7-T improved the performance of the photodetector in several ways. First, the use of poly-C_60_ reduced the dark current further by one order of magnitude (J_d_ = 5.7 × 10^−5^ mA cm^−2^ at −1 V; Table 1, E). Secondly, external quantum efficiency (EQE) measurements (Figure 3b) showed a small current contribution resulting from direct poly-C_60_ excitation in the wavelength region below 550 nm. This is much less, however, than the observed doubling of the short-circuit current when inserting poly-C_60_ (J_sc_ = 3.4 mA cm^−2^). This suggests that poly-C_60_ can assist in efficient charge separation between Cy7-T and TiO_2_ and prevents back recombination. Such a beneficial role of interfacial modifiers in hybrid solar cells incorporating both organic and inorganic materials has been demonstrated several times^29^,^30^.

The enhanced charge transfer process across the metal oxide – organic interface resulted also in a faster response speed of the photodiode. The temporal electrical response to nanosecond optical excitation at zero applied bias was used to probe photogenerated carrier extraction. The response decayed over ~2 μs when omitting poly-C_60_ (Figure 3c), but light detection was well accomplished within 1 μs when adding poly-C_60_, potentially allowing for applications requiring response frequencies up to 1 MHz. Optimized non-transparent and transparent devices (Table 1, E and F) showed very similar dark current values. Replacing the highly reflective back metal contact with a transparent electrode resulted in a lower J_sc_ value, as expected. Finally, we were able to fabricate photodetectors with larger active area without significant performance losses (Table 1, G). Large-area organic photodetectors have promising applications in X-ray medical imaging or industrial quality control, particularly because of the potential reductions in the costs when producing devices of several square centimeter^31^.

To characterize the photodiode sensitivity, we determined the specific detectivity D* (in units of cm Hz^½^ W^−1^ or Jones). D* is given as (A Δf)^½^ R/J_n_, where A is the effective area of the detector in cm^2^, Δf the electrical bandwidth in Hz, R the responsivity in A W^−1^, and J_n_ the noise current in A. With the assumption that under reverse bias the shot noise from the dark current is the dominant contribution to the overall electronic noise of the device^1^,^3^,^4^,^9^, the specific detectivity can be expressed as D* = R/(2q J_d_)^½^, where q is the absolute value of the electron charge and J_d_ is the dark current density (Table 1). The responsivity is given by R = J_ph_/I_light_, where J_ph_ is the photocurrent and I_light_ is the incident light intensity. To determine R, EQE values were measured under short-circuit conditions and under reverse bias (Figure 3b). The maximum EQE (at λ = 850 nm) increased from 13% electron per photon at zero bias to 23% at −2 V. Correspondingly, R increased with reverse bias to a maximum value of R = 165 mA W^−1^ at −2 V (Figure 3d). Specific detectivities were calculated based on the responsivity values and the dark current values (Figure 3d). D* monotonically decreased with increasing reverse bias from D* = 3 × 10^12^ Jones at −0.1 V to 1 × 10^12^ Jones at −2 V. The trend of D* demonstrates that the key to obtaining a high detector sensitivity is a high responsivity while keeping the dark current low.

We note that specific detectivities were calculated using responsivity values that were measured using light intensities in the ~0.1 mW cm^−2^ range. The linearity of the photodetector's responsivity was addressed by measuring the photocurrent for different light intensities (Supplementary Information S4). We applied light intensities ranging from 10^2^–10^−3^ mW cm^−2^ and measured a linear response of the photocurrent. Care must be taken when organic photodetectors are used to detect much lower light intensities, such as a few nW cm^−2^ in medical X-ray imaging^31^. This is because of the density of charge traps that is usually higher than in inorganic semiconductor materials. Trapped charges cause enhanced recombination and are extracted at a reduced speed from the organic material, deteriorating the responsivity and the bandwidth of the photodetector. These effects become important when the charge density generated by low-level light conditions is comparable to the charge trap density.

The figure of merits of our photodetector at λ = 850 nm, such as photoconversion efficiencies of 13%–23% and specific detectivities of ~10^12^ cm Hz^½^ W^−1^, compare favourably with results presented so far for selective NIR organic photodiodes^9^,^10^. Ultimately, the required level of device transparency and photometric perception will depend on the type of application. For example, a dual wavelength range specific photodetector can be envisioned by stacking a transparent NIR detector with a detector that selectively senses visible light. In that case the required transparency would be relatively low, because the combined photodetector appears coloured or grey, respectively. On the other hand, reasonable transparency perception and good colour rendering properties will be required for display or window-integrated applications.

## Methods

Photodetectors were fabricated in a glove box under N_2_ atmosphere (H_2_O < 1 ppm, O_2_ < 10 ppm). TiO_2_ films were prepared on cleaned ITO glass substrates (Geomatec, resistivity ~11 Ohms square^−1^) via a sol-gel process using titanium iso-propoxide as precursor^16^. Spin coated TiO_2_ films were heated within 3 h to 460°C in air, were kept for 2 h at that temperature, and were then cooled to room temperature. Before deposition of the active layers, TiO_2_ coated substrates were heated for 10 min at 120°C inside the glove box. Cy7-T was synthesized as described^17^ and spin coated as a 20 nm thick film from chlorobenzene solution. Layers of C_60_ (SES Research, 99.5% or 99.9%), MeO-TPD (Sigma-Aldrich, 98%) and MoO_3_ (Sigma Aldrich, 99.99%) were deposited by thermal evaporation (<5 × 10^−6^ mbar). C_60_ was photopolymerized under illumination (100 mW cm^−2^, N_2_) for 12 h. Ag (Cerac, 99.99%) and Au (Kurt J. Lesker, 99.99%) was evaporated through a shadow mask to define devices with active areas of 3.1 mm^2^, 7.1 mm^2^, or 1.6 cm^2^.

Absorption and transmission spectra were measured on a Varian Cary 50 UV-vis spectrophotometer. For the transmission spectrum shown in Figure 1d, glass was defined as the baseline. For the spectrum shown in Figure 2b, air was defined as the baseline. Film thicknesses were determined by profilometry (Ambios XP1). White light J-V characteristics were measured using 100 mW cm^−2^ simulated AM1.5G solar irradiation on a calibrated solar simulator from Spectra-Nova. EQE was measured using a monochromator and the light from a 300 W Xe lamp together with an AM1.5G filter set. The monochromatic light intensity was determined using a calibrated Si-diode. Bias on the device was applied using a Keithley 2400 sourcemeter. Photodiodes were characterized via illumination through the glass/ITO side only. For dark current measurements, devices were serially connected with resistors and currents were recorded using a Keithley 2000 multimeter by measuring the voltage drop.

Transient photocurrent experiments were carried out using a frequency-tripled Q-switched Nd:YAG laser (Ekspla NT-342) running at 20 Hz repetition rate. The excitation wavelength at 850 nm was generated by an optical parametric oscillator (idler output, 5 ns FWHM) and attenuated by grey filters (0.5–220 μJ cm^−2^). Temporal responses (photocurrent into 50–500 Ω loads) were measured with no bias applied on an oscilloscope (Tektronix DPO 7104C). Full lines in Figure 3c correspond to the best exponential fit.

Energy values shown in Figure 1b were taken from the literature: ITO^32^, TiO_2_^33^, Cy7-T^17^, MeO-TPD^34^, Au^35^ and MoO_3_^24^. For poly-C_60_, values from C_60_^36^ were adopted. The thickness of the transparent Au/MoO_3_ electrode was optimized using the optical model implemented in Setfos (www.fluxim.ch). Optical constants were taken from the literature: ITO^37^, TiO_2_^38^, MeO-TPD^39^, MoO_3_^40^ and for Au from the Setfos database. For poly-C_60_, values from C_60_^41^ were adopted. Optical constants for Cy7-T were determined by spectroscopic ellipsometry (Supplementary Information S5).

## Author Contributions

Devices were fabricated by H.Z., the dye was synthesized by A.C.V., transient spectroscopy was measured by J. De J., ellipsometry measurements were carried out by S.J., R.H., R.S. and F.N. contributed to project planning and manuscript preparation.

## Supplementary Material

Supplementary Information Supplementary Information

## Figures and Tables

**Table 1:** Current-voltage characteristics of ITO/TiO_2_(50 nm)/poly-C_60_/Cy7-T(20 nm)/MeO-TPD/MoO_3_(30 nm)/(top electrode) photodiodes

Devicea)	poly-C_60_ (nm)	MeO-TPD (nm)	J_d_ at 0 Vb) (mA cm^−2^)	J_d_ at −1 V (mA cm^−2^)	R_sh_ (kΩ cm^2^)	R_s_ (Ω cm^2^)	J_sc_ (mA cm^−2^)
A	0	0	1.9 × 10^−3^	4.3 × 10^−1^	3.9	5	2.73
B	0	10	7.1 × 10^−4^	5.7 × 10^−2^	20	40	2.01
C	0	40	4.9 × 10^−7^	5.5 × 10^−4^	2 × 10^3^	53	1.70
D	0	100	1.4 × 10^−8^	3.6 × 10^−4^	4 × 10^3^	400	0.50
E	9	40	3.2 × 10^−7^	5.7 × 10^−5^	28 × 10^3^	83	3.40
Fc)	9	40	3.7 × 10^−7^	3.3 × 10^−5^	30 × 10^3^	63	1.99
G	9	40	2.7 × 10^−7^	6.9 × 10^−5^	20 × 10^3^	260	2.05

^a)^The top electrode for devices A–E was 80 nm Ag, for the transparent devices F and G gold (8 nm)/MoO_3_ (40 nm). The device area was 3.1 mm^2^ for A–F, and 1.6 cm^2^ for G;

^b)^J_d_ denotes the dark current, R_sh_ the shunt resistance, R_s_ the series resistance, and J_sc_ the short-circuit current density measured at 100 mW cm^−2^ simulated AM1.5G solar irradiation;

^c)^Rectification of the dark current at ±1 V was 6.6 × 10^4^.
