# Supplementary material for: Transparent Organic Photodetector using a Near-Infrared Absorbing Cyanine Dye
Source: Sci Rep. 2015 Mar 24;5:9439. doi: 10.1038/srep09439 (PMC4371738; doi:10.1038/srep09439)
Supplement: Supplementary Information [file srep09439-s1.pdf]

Supplementary Information

Transparent Organic Photodetector using a Near-Infrared Absorbing Cyanine Dye

Hui Zhang, Sandra Jenatsch, Jelissa De Jonghe, Frank Nüesch, Roland Steim, Anna C. Véron, and Roland Hany*

Supplementary Information 1

The growth mode of thin metal films depends on the metal type, substrate, film deposition method, or temperature. Large scale coalescence of metal islands during deposition eventually forms a continuous layer that allows for electrical conduction over macroscopic distances. The best compromise between increasing conductivity and decreasing transparency is close to that coalescence point. For Ag deposited on MoO_3, sheet resistance values of ~45 Ω $square^{-1}$ (8 nm Ag) or ~10 Ω $square^{-1}$ (10 nm Ag) were measured[S11]. In reference S12, these sheet resistance values were reported as ~95 Ω $square^{-1}$ (8 nm Ag) and ~40 Ω $square^{-1}$ (12 nm Ag). The conductivity for thin Au films on MoO_3 seems to be even slightly higher, and sheet resistances <10 Ω $square^{-1}$ were measured for 10 nm thick Au films[S13]. In all cases, sheet resistances decreased with increasing metal film thickness. We calculated AVT values of photodetectors with different thickness combinations of the top Au / MoO_3 electrode (Figure S1). For a 40 nm thick MoO_3 layer, AVT values are 71.5% and 68% for Au thicknesses of 8 nm and 12 nm, respectively. This indicates that when the electrode sheet resistance value needs to be decreased, this can be accepted with a relatively small drop in the AVT .

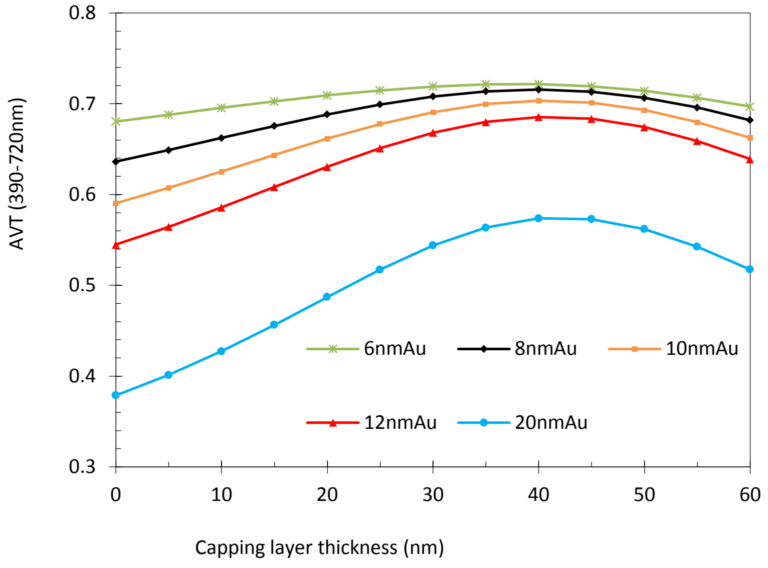

Figure S1 | Calculated average visible transmittance (AVT) values for different thickness combinations of the top Au / MoO_3 electrode.

S11. Schubert, S., Hermenau, M., Meiss, J., Müller-Meskamp, L. & Leo, K. Oxide sandwiched metal thin-film electrodes for long-term stable organic solar cells, *Adv. Funct. Mater.* **22**, 4993-4999 (2012).

S12. Tian, B., Williams, G., Ban, D. & Aziz, H. Transparent organic light-emitting devices using a MoO_3/Ag/MoO_3 cathode, *J. Appl. Phys.* **110**, 104507 (2011).

S13. Wrzesniewski, E., Eom, S.-H., Hammond, W. T., Cao, W. & Xue, J. Transparent oxide/metal/oxide trilayer electrode for use in top-emitting organic light-emitting diodes, *J. Photonics for Energy* **1**, 011023 (2011).

Supplementary Information 2

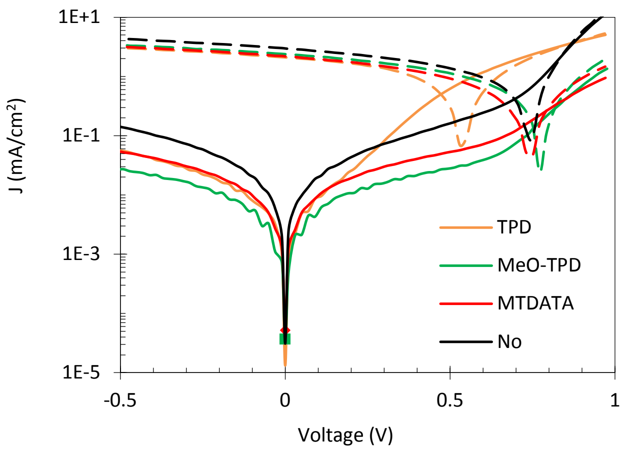

Figure S2 | Current-voltage characteristics in the dark (full lines) and under illumination (dotted lines, 100 mW cm^{-2}) of ITO / TiO_2(50 nm) / Cy7-T(20 nm) / EBL(10 nm) / MoO_3(30 nm) / Ag(80 nm) devices using different electron-blocking layers EBL. m-MTDATA is 4,4,4',4'-Tris[(3-methylphenyl) phenylamino] triphenylamine (Sigma-Aldrich, ≥99.0%), TPD is N,N'-Bis(3-methylphenyl)-N,N'-diphenylbenzidine (Sigma-Aldrich, 99%).

Table S2 | Device performance / parameters for J-V curves shown in Figure S2.

EBL[a)]				Device performance				
	HOMO (eV)	LUMO (eV)	Hole mobility (cm^2 V^{-1} s^{-1})	V_{oc} (V)	J_{sc} (mA cm^{-2})	Eff (%)	FF (%)	$r_{p/d}$ [b)] (at -0.1 V)
no				0.74	2.73	0.75	37	1.5×10^2
m-MTDATA	-5.1	-2.0	3×10^{-5}	0.74	2.20	0.50	31	2.2×10^2
TPD	-5.4	-2.4	1.4×10^{-3}	0.51	2.16	0.40	37	2.6×10^2
MeO-TPD	-5.1	-1.9	1.1×10^{-3}	0.77	2.01	0.50	32	4.9×10^2

[a)] HOMO, LUMO energies and hole mobility values were taken from

He, G., Walzer, K., Pfeiffer, M., Leo, K., Pudzich, R. & Salbeck, J. *Proceedings of SPIE* **5519**, 42-47 (2004);

Kulshreshtha, C., Choi, J. W., Kim, J.-k., Jeon, W. S., Suh, M. C., Park, Y. & Kwon, J. H. *Appl. Phys. Lett.* **99**, 023308 (2011);

Shirota, Y. *J. Mater. Chem.* **10**, 1-25 (2000);

Lee, J., Kim, S.-Y., Kim, C. & Kim, J.-J. *Appl. Phys. Lett.* **97**, 083306 (2010);

Goushi, K., Yoshida, K., Sato, K. & Adachi, G. *Nature Photon.* **6**, 253-258 (2012).

[b)] $r_{p/d}$ is the ratio between the photocurrent and the dark current.

We observed that for comparable J_{sc} current values, the dark current was smallest when using MeO-TPD, resulting in the largest value for $r_{p/d}$.

Supplementary Information 3

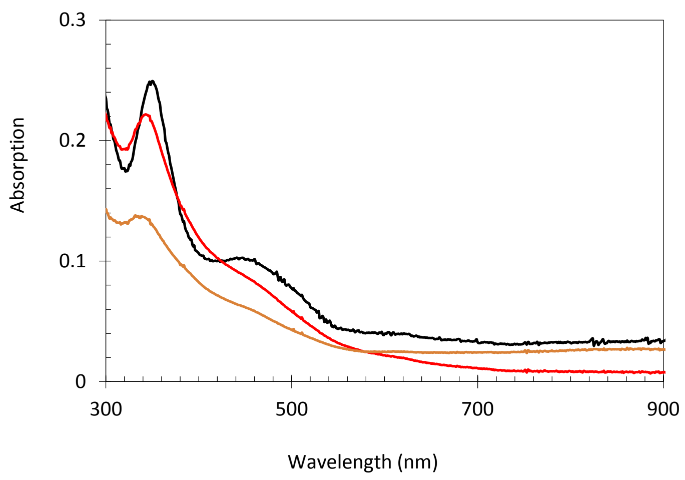

Figure S3 | Absorption spectra of a ~15 nm thick C_{60} film after evaporation (black line), after illumination (100 mW cm^{-2}) for 12 h under nitrogen atmosphere (red line), and after spin coating with chlorobenzene (orange line). During light exposure C_{60} polymerizes [S3] and becomes partially insoluble in chlorobenzene. The C_{60} film thickness after solvent treatment is ~ 9 nm.

S3. Zhang, H., Borgschulte, A., Castro, F. A., Crockett, R., Gerecke, A. C., Deniz, O., Heier, J., Jenatsch, S., Nüesch, F., Sanchez-Sanchez, C., Zoladek-Lemanczyk, A. & Hany, R. *Adv. Energy Mater*. **5**, 1400734 (2014).

Supplementary Information S4

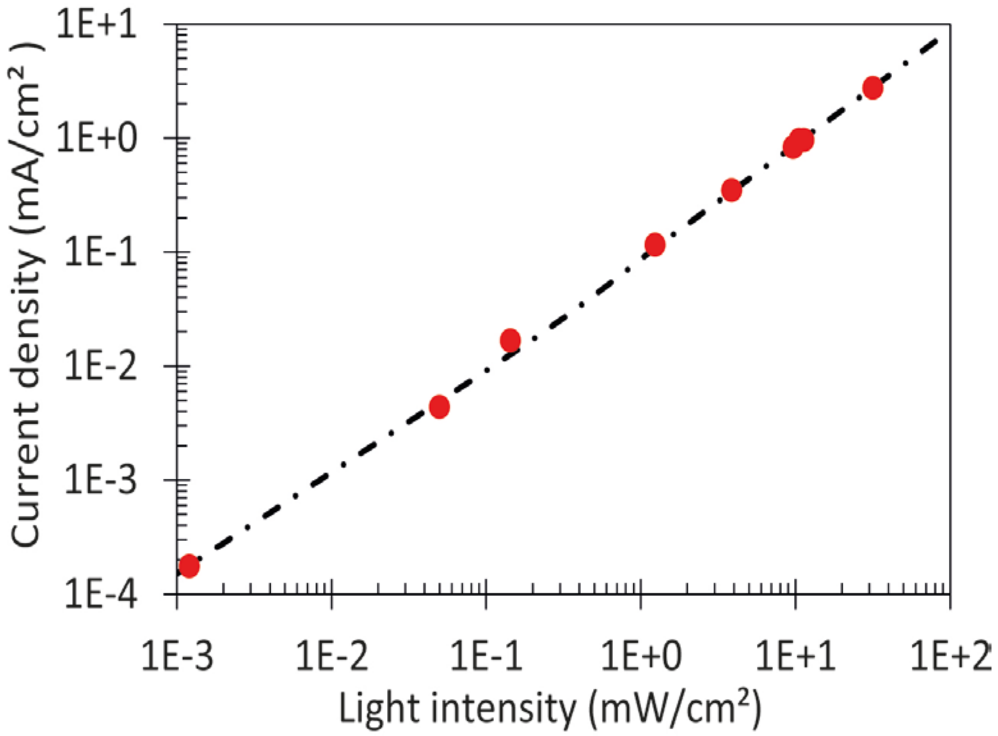

Figure S4 | Photocurrent versus light intensity (wavelength range 780 – 880 nm).

Supplementary Information S5

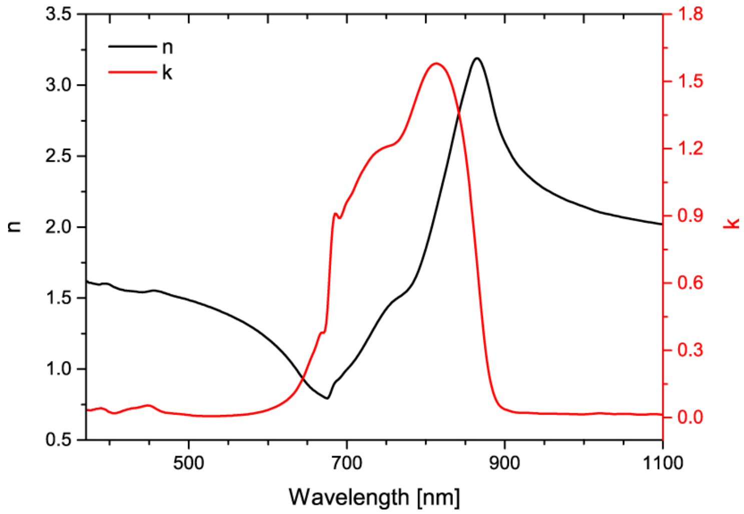

Figure S5 | Refractive index (n) and extinction coefficient (k) of Cy7-T. Optical constants for Cy7-T were determined by spectroscopic ellipsometry (M-2000, J.A. Woolam Co., Inc.). For the ellipsometry measurements, cyanine films were spin coated from chlorobenzene onto silicon wafers.
